# P02.64. Vitamin D sufficiency is necessary for integrative treatment-associated improvements in chronic pain status

**DOI:** 10.1186/1472-6882-12-S1-P120

**Published:** 2012-06-12

**Authors:** G Plotnikoff, J Dusek

**Affiliations:** 1 Penny George Institute for Health and Healing, Minneapolis, USA

## Purpose

To examine the relationship between improved vitamin D status and reported severity of chronic pain.

## Methods

Prospective observational study over 12 weeks of 252 patients with pain of any source lasting more than 6 months who sought integrative medicine evaluation and treatment at one of 9 Bravenet clinical sites across the United States. Interventions were not mandated. Baseline vitamin D status was reported to each clinician. Treatment options included conventional therapies as well as acupuncture, nutrition, massage and mind-body skills training. Measurements included the Brief Pain Inventory and 25-OH-vitamin D status.

## Results

A total of 252 adults met eligibility criteria. Mean 25-OH-vitamin D levels and standard deviations at the start and 12 weeks later for all participants were 33.43 (17.05) and 39.58 (16.29). (p <0.0001). The subset of low back pain patients demonstrated similar values of 32.71 (14.34) increasing after 12 weeks to 39.19 (13.26) (p <0.0001). Of all participants, 153 (60.7%) achieved 25-OH-vitamin D levels above the 2010 international recommendation of ≥ 30 ng/ml and 99 (39.3%) did not. Of all 136 back pain participants, 90 (66.2%) achieved levels ≥ 30 ng/ml and 46 (33.8%) did not. Median average pain scores decreased from 5 to 4 during the study. Regardless of the integrative therapies applied, achievement of a serum level ≥ 30 ng/ml was necessary for significant improvement in average pain (p =0.0018 for all patients, p =0.022 for all back pain).

## Conclusion

Chronic pain patients who sought integrative medicine demonstrated a high incidence of vitamin D deficiency. In the setting of additional treatments, serum levels ≥ 30 ng/ml were required for significant improvement in average pain scores. Failure to achieve a serum level ≥ 30 ng/ml represents a confounder of any therapeutic intervention in both clinical practice and clinical trials for chronic pain including chronic low back pain.

